# Effect of body mass index on response to neo-adjuvant therapy in HER2-positive breast cancer: an exploratory analysis of the NeoALTTO trial

**DOI:** 10.1186/s13058-020-01356-w

**Published:** 2020-10-27

**Authors:** Serena Di Cosimo, Luca Porcu, Dominique Agbor-tarh, Saverio Cinieri, Maria Alice Franzoi, Maria Carmen De Santis, Cristina Saura, Jens Huober, Debora Fumagalli, Miguel Izquierdo, Martine Piccart, Maria Grazia Daidone, Evandro de Azambuja

**Affiliations:** 1 Biomarkers Unit, Department of Applied Research and Technological Development, Fondazione IRCCS Istituto Nazionale dei Tumori, via G.A. Amadeo 42, 20133 Milano, Italy; 2 Laboratory of Methodology for Clinical Research, Department of Oncology, Istituto di Ricerche Farmacologiche Mario Negri IRCCS, Milano, Italy; 3 Frontier Science (Scotland) Ltd, Kincraig, UK; 4 San Antonio Perrino Hospital, Brindisi, Italy; 5 Institut Jules Bordet and l’Universitè Libre de Bruxelles (U.LB), Brussels, Belgium; 6 Radiation Oncology, Fondazione IRSCCS Istituto Nazionale dei Tumori, Milano, Italy; 7 Vall d’Hebron Institute of Oncology, Barcelona, Spain; 8 University of Ulm, Ulm, Germany; 9 Breast International Group (BIG), Boulevard de Waterloo 76, 1000 Bruxelles, Belgium; 10 Oncology Clinical Development, Oncology Business Unit, Novartis Pharma AG, Basel, Switzerland

**Keywords:** Body mass index, Neo-adjuvant treatment, HER2-positive breast cancer, Pathological complete response

## Abstract

**Background:**

Obesity is a risk factor for breast cancer (BC) development, recurrence, and death. In view of this, we aimed to investigate the clinical value of obesity in BC patients treated with anti-HER2 therapies in the NeoALTTO trial, which randomized 455 patients to neo-adjuvant lapatinib, trastuzumab, or their combination plus paclitaxel.

**Methods:**

Patients were classified according to their basal body mass index (BMI) into underweight (< 18.5 kg/m^2^), normal (≥ 18.5; < 25 kg/m^2^), overweight (≥ 25; < 30 kg/m^2^), and obese (≥ 30 kg/m^2^) WHO categories. Univariate and multivariate logistic regression analyses were performed using BMI as a categorical variable. Pathological complete response (pCR) and event-free survival (EFS) were the NeoALTTO primary and secondary outcomes, respectively.

**Results:**

Among 454 patients analyzed, 14 (3%), 220 (48%), 137 (30%), and 83 (18%) were classified as underweight, normal weight, overweight, and obese, respectively; 231 (51%) and 223 (49%) had hormone receptor (HR)-positive and HR-negative primary tumors; 160 (35%) achieved pCR. In the overall patient population, no association was found between BMI groups and pCR, as we reported pCR rates of 57.1%, 35%, 30.7%, and 39.8% in underweight, normal weight, overweight, and obese cases, respectively. In contrast, in HR-positive tumors, overweight or obesity was generally associated with decreased likelihood of achieving a pCR independently of other clinical variables, including planned surgery, nodal status, and tumor size (odds ratio [OR] = 0.55, 95%CI 0.30–1.01, as compared to normal or underweight; *p* = 0.053); notably, no differential effect of BMI with respect to pCR was observed in HR-negative cases (odds ratio [OR] = 1.30, 95%CI 0.76–2.23, as compared to normal or underweight; *p* = 0.331), resulting in a statistically significant interaction between BMI and HR status (*p* = 0.036). There was no association between BMI and EFS neither in the overall nor in the HR-positive population, but this analysis was under-powered.

**Conclusions:**

NeoALTTO patients overweight or obese at baseline and with HR-positive primary BC appeared less likely to achieve pCR after neo-adjuvant anti-HER2 therapies. This finding paves the way to future research in targeting the interplay between HER2/HR signaling and metabolism.

## Introduction

Obesity is one of the most common public health problems worldwide, and its incidence is increasing steadily over the past two decades in both developed and developing countries. Epidemiological data confirm that obesity is independently associated with an increased incidence of various solid tumors, including breast cancer (BC), and is a poor prognostic factor in early and metastatic BC patients [1, 2].

Despite its impact on BC development and prognosis, few studies have investigated the predictive value of obesity for response to systemic therapies mostly focusing on unselected BC patient populations [3]. Hence, it is not surprising that the association between obesity and treatment response is still controversial.

In HER2-positive early BC patients, no significant difference was reported between obese and non-obese patients treated in the N9831 adjuvant trial, which compared anthracycline/taxane-based regimen versus its combination with trastuzumab, as the trend toward decreased disease-free survival observed in obese patients treated with chemotherapy alone was reverted by combining chemotherapy with trastuzumab [4]. In the neo-adjuvant setting, the relationship between obesity and response to anti-HER2 agents has been evaluated mainly by retrospective or institutional series, which reported that obesity can independently and negatively affect response rate, including pathological complete response (pCR) [5–7].

Here, we sought to analyze if the relationship between obesity and adverse clinical and pathological characteristics, primary treatment response, and disease outcome in HER2-positive early BC patients treated in the setting of a large prospective randomized clinical trial such as NeoALTTO [8].

## Materials and methods

Details of the NeoALTTO (Breast International Group 1-06) trial have been already reported [8]. Briefly, the study was a randomized, multicenter, open-label, phase III trial evaluating lapatinib (L), trastuzumab (T), or their combination (L+T) with paclitaxel as neo-adjuvant therapy in 455 patients with HER2-positive primary BC > 2 cm. Primary objective was the rate of pathological complete response (pCR), defined as the absence of residual invasive cancer in breast surgical specimens (ypT0/is). Secondary endpoints included event-free survival (EFS) as defined per protocol. The present study tested whether as compared to normal and/or underweight, obese/overweight HER2-positive BC patients were less likely to attain pCR overall and/or with respect to other clinical variables, including primary tumor hormone receptor (HR) status. The hypothesis was that obesity/overweight would be associated with reduced response to L, T, and L+T plus paclitaxel and that this detrimental effect could be more prominent in HR-positive cases.

### Statistical analysis

BMI was calculated as baseline weight in kilograms divided by the square of height in meters (kg/m^2^), and groups were separated into underweight (< 18.5 kg/m^2^), normal (≥ 18.5; < 25 kg/m^2^), overweight (≥ 25; < 30 kg/m^2^), and obese (≥30 kg/m^2^) according to the WHO classification [9]. To test interaction with the HR status, BMI was dichotomized into underweight/normal vs overweight/obese. The association between BMI and other clinical and pathological characteristics at baseline was evaluated using Pearson’s chi-squared test and Kruskal-Wallis test for categorical and continuous variables, respectively. The impact of the covariates on pCR was modeled with logistic regression. In multivariable logistic regression models, odds ratios (ORs) were adjusted by primary tumor size, nodal status, and planned surgery. Results were presented as ORs with 95% confidence intervals (CIs). Median (IQR) follow-up was estimated using the reverse Kaplan-Meier method. Survival curves were estimated by the Kaplan-Meier method. Survival outcomes were analyzed using the Cox proportional hazards model. All analyses were performed using SAS 9.4 software (Copyright (c) 2016 by SAS Institute Inc., Cary, NC, USA).

## Results

### Patient characteristics

All but one NeoALTTO patients (*n* = 454) had known BMI before starting neo-adjuvant therapy and were included in the present analysis. Patient and tumor characteristics overall and according to BMI are depicted in Table 1. Patients were categorized as underweight, normal, overweight, and obese in 14 (3%), 220 (48%), 137 (30%), and 83 (18%) cases, respectively. No significant association was found between BMI and other clinical features, with the unique exception of menopausal status and age. Obese and overweight patients were in fact more likely to be postmenopausal and older as compared to under/normal weight patients [postmenopausal status, 54% and 60% versus 36% and 40%, respectively, *p* = 0.036; median age (years), 52 and 51 years versus 46 and 48 years, respectively, *p* < 0.001]. A slightly increased number of obese and overweight patients were also reported in dual as compared to each single agent arms: 79 (36%) in L+T, 69 (31%) in L, and 72 (33%) in T arms (*p* = 0.099). No association was found between BMI and tumor stage (*p* = 0.887), grade (*p* = 0.121), and hormone receptor (HR) status (*p* = 0.112).

**Table 1:** NeoALTTO patient characteristics by body mass index (BMI) categories

	Overall (***N*** = 454)	BMI categories				
		Underweight (***N*** = 14)	Normal weight (***N*** = 220)	Overweight (***N*** = 137)	Obese (***N*** = 83)	***p*** value
**Median age (years) at baseline**	50	46	48	51	52	**< 0.001**
**Randomized arm,** ***N*** **(%)**						
L	153 (34)	1 (7)	83 (38)	49 (36)	20 (24)	0.099
T	149 (33)	6 (43)	71 (32)	40 (29)	32 (39)	
L + T	152 (33)	7 (50)	66 (30)	48 (35)	31 (37)	
**Clinical tumor size,** ***N*** **(%)**						
≤ 5 cm	274 (60)	9 (64)	135 (61)	79 (58)	51 (61)	0.887
> 5 cm	180 (40)	5 (36)	85 (39)	58 (42)	32 (39)	
**HR status,** ***N*** **(%)**						
Negative	223 (49)	3 (21)	110 (50)	73 (53)	37 (45)	0.112
Positive	231 (51)	11 (79)	110 (50)	64 (47)	46 (55)	
**Menopausal status,** ***N*** **(%)**						
Post-menopausal	189 (42)	5 (36)	76 (35)	68 (50)	40 (48)	**0.036**
Pre-menopausal	219 (48)	9 (64)	122 (55)	53 (39)	35 (42)	
NA and age < 50	14 (3)	0	9 (4)	2 (1)	3 (4)	
NA and age ≥ 50	32 (7)	0	13 (6)	14 (10)	5 (6)	
**Histology grade,** ***N*** **(%)**						
G1	12 (3)	1 (7)	2 (< 1)	5 (4)	4 (5)	0.121
G2	171 (38)	8 (57)	91 (41)	48 (35)	24 (29)	
G3	205 (45)	3 (21)	101 (46)	62 (46)	39 (47)	
GX	65 (14)	2 (14)	26 (12)	21 (15)	16 (19)	
**Planned surgery,** ***N*** **(%)**						
Conservative surgery	130 (29)	5 (36)	63 (29)	39 (28)	23 (28)	0.944
Mastectomy	324 (71)	9 (64)	157 (71)	98 (72)	60 (72)	
**Clinical nodal status,** ***N*** **(%)**						
N0/1	382 (84)	13 (93)	189 (86)	112 (82)	68 (82)	0.531
≥ N2, Nx or missing	72 (16)	1 (7)	31 (14)	25 (18)	15 (18)	

*HR* hormone receptor, *BMI* body mass index, *L* lapatinib, *T* trastuzumab, *L+T* lapatinib + trastuzumab, *NA* non-available

### Association between BMI and pCR

**Fig. 1:**
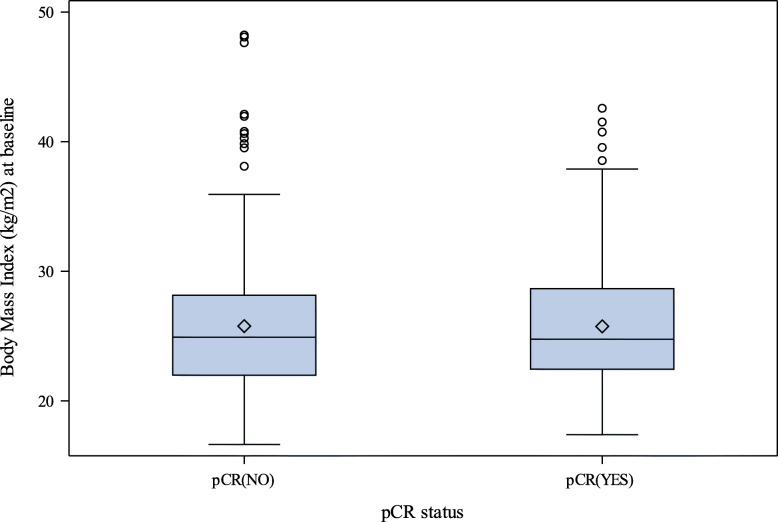
Box plot of body mass index in NeoALTTO patients attaining or not pathological complete response (pCR)

Overall, 160 patients (35%) achieved a pCR at the time of surgery. The pCR rates in underweight, normal, overweight, and obese groups were 8/14 (57%), 77/220 (35%), 42/137 (31%), and 33/83 (40%), respectively. BMI did not significantly differ between patients with pCR and those with residual disease at surgery. Specifically, according to the median value, the measurements of BMI were similar among patients achieving or not pCR, median (IQR) BMI, 24.8 (22.5–28.7) kg/m^2^ and 24.9 (22.0–28.2) kg/m^2^ respectively (Fig. 1). As shown in Table 2, univariate logistic regression analysis confirmed that BMI measurements were not associated with pCR [OR underweight vs normal, 2.48 (95%CI 0.83–7.39); OR overweight vs normal, 0.82 (95%CI 0.52–1.30); OR obese vs normal, 1.23 (95%CI 0.73–2.06); *p* = 0.191). Point and interval estimates of ORs were substantially confirmed in multivariate analysis. As continuous variable, BMI had no discriminatory capability (AUC = 0.50). In subgroup analysis by treatment arm, BMI was not associated to pCR in all treatment arms (Supp. Table 1).

**Table 2:** Effect of body mass index (BMI) on the rate of pathological complete response (pCR)

BMI with 4 categories	pCR (%)	***N***	Univariate OR (95%CI)	Univariate ***p*** value	Multivariate^**^**^ OR (95%CI)	Multivariate ***p*** value
BMI at baseline	160 (35.2)	454				
Normal weight	77 (35.0)	220	–	–	–	–
Underweight	8 (57.1)	14	2.48 (0.83–7.39)	0.104	3.30 (1.07–10.12)	**0.037**
Overweight	42 (30.7)	137	0.82 (0.52–1.30)	0.398	0.78 (0.49–1.25)	0.309
Obese	33 (39.8)	83	1.23 (0.73–2.06)	0.443	1.28 (0.75–2.18)	0.362

*BMI* body mass index, *pCR* pathological complete response, *OR* odds ratio, *CI* confidence interval

^^^Adjusted for planned surgery, HR, nodal status, and tumor size

### BMI and pCR by hormone receptor status

While there was no evidence that BMI was an independent factor influencing the pCR rate in the overall NeoALTTO patient population, overweight/obese patients with HR-positive primary tumor were less likely to achieve pCR as compared to normal/underweight counterparts (OR 0.56, 95%CI 0.31–1.01; *p* = 0.054; Table 3). Notably, this was not the case for HR-negative cases (OR 1.31, 95%CI 0.77–2.22; *p* = 0.324), resulting in a statistically significant interaction between BMI and HR status (*p* = 0.036). The effect of baseline BMI on the rate of pCR in HR-positive cases remained worth of consideration even after adjusting for other relevant clinical variables, including tumor size, nodal status, and planned surgery (OR 0.55, 95%CI 0.30–1.01; *p* = 0.053).

**Table 3:** BMI and hormone receptor status as predictive factors of pCR

			Univariate analysis		Multivariate analysis^**°**^	
BMI categories	***N***	pCR (%)	OR (95%CI)	***p*** value	OR (95%CI)	***p*** value
**HR-positive population**	231	62 (26.8)				
Underweight/normal	121	39 (32.2)	1		1	
Overweight/obese	110	23 (20.9)	0.56 (0.31–1.01)	0.054	0.55 (0.30–1.01)	0.053
**HR-negative population**	223	98 (43.9)				
Underweight/normal	113	46 (40.7)	1	0.324	1	0.331
Overweight/obese	110	52 (47.3)	1.31 (0.77–2.22)		1.30 (0.76–2.23)	
			*p value HR+* vs *HR−*	**< 0.0001**		
			***p value for interaction***	**0.036**		

*BMI* body mass index, *pCR* pathological complete response, *HR* hormone receptor, *OR* odds ratio, *CI* confidence interval

^°^Adjusted for planned surgery, nodal status, and tumor size

### Association between BMI and EFS

After a median follow-up of 6.7 years (IQR 5.8–6.8), 127 (28.0%) EFS events were observed. Death occurred in 77 (17.0%) patients. Baseline BMI, either as a continuous or a categorical variable, was not significantly associated with EFS (overall or in the HR-positive cohort) (Supp. Fig. 1). Of note, this analysis was underpowered.

## Discussion

The neo-adjuvant setting best suits the purpose of investigating new treatments and potential factors influencing response. In addition, pCR represents an important surrogate marker for favorable prognosis in HER2-positive breast cancer patients [10]. In this work, we investigated the impact of BMI on pCR following treatment with L, T, and their combination plus paclitaxel in HER2-positive BC patients enrolled in the large randomized phase III study NeoALTTO. Half of the study patient population had a BMI in the healthy 18.5–25 range and reported a pCR rate of 35%. Notably, underweighted cases (BMI < 18.5), although few, appeared almost twice as likely to attain a pCR. Patients with a BMI < 18.5 are generally underrepresented in BC epidemiological [11] and clinical studies [3], at least in the western world countries. The scenario is different in Asia where up to 7% of BC cases are newly diagnosed in underweighted patients [12]. Whether the observed effect of underweight on pCR rate is genuine needs further investigation in pooled analysis sufficiently large to allow adjustment for confounding factors [11].

Notably, BMI was associated with decreased pCR rates in HR-positive but not in HR-negative cases, with a significant test for interaction. This result was found in the context of the anthracycline-free chemotherapeutic regimen of NeoALTTO and should be confirmed in other HER2 BC populations, where pCR rate are expected to be higher. Nevertheless, the data is not only in line with that reported with standard neoadjuvant chemotherapy [3], showing an effect of BMI on pCR rate limited to luminal breast cancer cases, but also consistent with previous studies, showing that a BMI above normal limits has negative impact on prognosis of HR-positive BC [3].

.The effect is particularly evident when treatment consists of aromatase inhibitors [13], though recently it has been also reported with fulvestrant [14]. This evidence may be explained by the endocrine role of adipose tissue, which is involved in the metabolism of female reproductive hormones. In fact, fatty cells have their own aromatase activity, which is a significant source of estrogens in particular in menopausal women. Moreover, insulin resistance is a frequent condition in obese patients, and an intense cross-talk between insulin and estrogen signaling pathways has already been demonstrated [15]. High levels of insulin and insulin-like growth factors (IGFs) may also play direct role in stimulating tumor proliferation in obese women, independently from sexual hormone pathways [15, 16].

Clinical trials are currently ongoing exploring combined insulin and IGF-1 receptors targeting, or simultaneous use of anti-IGF-1 and 2 antibodies [17, 18]. These strategies might be valuable to hamper both insulin and IGF signaling, and the compensatory mechanisms triggered by single receptor blockade, which may explain the failure of previous anti-IGF-1R development [17]. According to the upcoming results, we could foresee the potential of developing specific treatments for overweight/obese patients by combining such treatment with anti-HER2 therapies, in at least 20% of HER2-positive cases, which also co-express IGF-1R. However, it is important to mention that, in contrast to preclinical studies [19], a post hoc analysis of the N9831 study did not detect a difference in the benefit of adjuvant trastuzumab according to IGF1R protein status measured by immunohistochemistry [20]. Until more evidence becomes available, dietary and behavior modification, including increased aerobic and strength training exercise, are recommended to prevent weight gain, reduce biomarkers associated with inflammation and comorbidities, and improve lifestyle function status [21].

Overall, these data offer plausible biological reasons for the lower pCR rate observed in the luminal-like HER2-positive breast cancer patients with high BMI treated in the NeoALTTO. This is particularly interesting if we consider that HER2 has always been accounted as a factor of endocrine resistance in BC, with the capability of attenuating the effects of endocrine therapy [22], and suggests that hormonal metabolic pathway is still active in HER2-positive tumors and plays a role in determining the behavior of disease, even in presence of adequate HER2 blockade.

## Conclusion

In the NeoALTTO study, obesity and overweight are associated with reduced chance of attaining pCR in HER2-positive luminal BC patients. This finding paves the way to future research in developing combined neo-adjuvant strategies aimed at obtaining a complete blockade of driving growth factor signals for metabolism via both HER and hormone receptors.

## Supplementary information

**Additional file 1:** Supp. Table 1 pCR rate according to BMI categories and treatment arms.

**Additional file 2:** Supp. Figure 1 KM curve of Event free survival by BMI categories.

## Data Availability

Due to Informed Consent Form, data privacy, and Intellectual Property Rights-related restrictions, the clinical data cannot be made public, i.e., accessible for anyone, for any purpose without a review process and without putting an agreement in place. Nevertheless, raw data are available upon request and any requests can be directed to the central (Neo) ALTTO team.
