# Supplementary material for: Effect of body mass index on response to neo-adjuvant therapy in HER2-positive breast cancer: an exploratory analysis of the NeoALTTO trial
Source: Breast Cancer Res. 2020 Oct 27;22:115. doi: 10.1186/s13058-020-01356-w (PMC7590445; doi:10.1186/s13058-020-01356-w)
Supplement: Supplementary file 1 — Additional file 1: Supp. Table 1 pCR rate according to BMI categories and treatment arms. [file 13058_2020_1356_MOESM1_ESM.docx]

**Supp. Table1 - pCR rate according to BMI categories and treatment arms**

**LAPATINIB, N = 153**

| pCR  N, column % | **BMI categories** | | | |
| --- | --- | --- | --- | --- |
|  | **Underweight** | **Normal weight** | **Overweight** | **Obese** |
| **No** N  % | 1  100 | 62  74.7 | 41  83.7 | 11  55.0 |
| **Yes** N  % | 0  0 | 21  25.3 | 8  16.3 | 9  45.0 |

Pearson χ^2^ (3 d.f.) = 6.5967; p= 0.086

**TRASTUZUMAB, N = 149**

| pCR  N, column % | **BMI categories** | | | |
| --- | --- | --- | --- | --- |
|  | **Underweight** | **Normal weight** | **Overweight** | **Obese** |
| **No** N  % | 4  66.7 | 51  71.8 | 29  72.5 | 21  65.6 |
| **Yes** N  % | 2  33.3 | 20  28.2 | 11  27.5 | 11  34.4 |

Pearson χ^2^ (3 d.f.) = 0.5451; p= 0.909

**LAPATINIB + TRASTUZUMAB, N = 152**

| pCR  N, column % | **BMI categories** | | | |
| --- | --- | --- | --- | --- |
|  | **Underweight** | **Normal weight** | **Overweight** | **Obese** |
| **No** N  % | 1  14.3 | 30  45.5 | 25  52.1 | 18  58.1 |
| **Yes**  N  % | 6  85.7 | 36  54.5 | 23  47.9 | 13  41.9 |

Pearson χ^2^ (3 d.f.) = 4.9048; p= 0.179
