# Supplementary material for: Effect of body mass index on response to neo-adjuvant therapy in HER2-positive breast cancer: an exploratory analysis of the NeoALTTO trial
Source: Breast Cancer Res. 2020 Oct 27;22:115. doi: 10.1186/s13058-020-01356-w (PMC7590445; doi:10.1186/s13058-020-01356-w)
Supplement: Supplementary file 2 — Additional file 2: Supp. Figure 1 KM curve of Event free survival by BMI categories. [file 13058_2020_1356_MOESM2_ESM.docx]

**Supp. Fig.1 KM curve of Event free survival by BMI categories**

Legend: KM: Kaplan-Meier, BMI: body mass index, Underweight: BMI of <18.5kg/m^2^, Normal weight: BMI of 18.5- 24.9 kg/m^2^, Overweight BMI of 25-29.9mg/m^2^, Obese BMI ≥ 30mg/m^2^
